# Immunomodulation Mechanism of Antidepressants: Interactions between Serotonin/Norepinephrine Balance and Th1/Th2 Balance

**DOI:** 10.2174/157015912800604542

**Published:** 2012-06

**Authors:** Matteo Martino, Giulio Rocchi, Andrea Escelsior, Michele Fornaro

**Affiliations:** 1 Department of Neuroscience, Section of Psychiatry, University of Genova, Genoa, Italy; 2 Scienze della Formazione, University of Catania, Catania, Italy

**Keywords:** Antidepressants, cytokines, neurotransmitters, receptors, Th1, Th2.

## Abstract

Neurotransmitters and hormones regulate major immune functions, including the selection of T helper (Th)1 or Th2 cytokine responses, related to cell-mediated and humoral immunity, respectively. A role of imbalance and dynamic switching of Th1/Th2 system has been proposed, with relative displacement of the immune reserve in relation to complex interaction between Th1/Th2 and neuro-hormonal balance fluctuations, in the pathogenesis of various chronic human diseases, probably also including psychiatric disorders. Components of the stress system such as norepinephrine (NE) and glucocorticoids appear to mediate a Th2 shift, while serotonin (5-HT) and melatonin might mediate a Th1 shift. Some antidepressants would occur affecting these systems, acting on neurotransmitter balance (especially the 5-HT/NE balance) and expression levels of receptor subtypes, which in turn affect cytokine production and relative Th1/Th2 balance. It could be therefore hypothesized that the antidepressant-related increase in NE tone enhances the Th2 response, while the decrease in NE tone or the increase in 5-HT tone enhances the Th1 response. However, the neurotransmitter and Th1/Th2 balance modulation could be relative, aiming to restore physiological levels a previous imbalance in receptor sensitivity and cytokine production. The considerations on neuro-immunomodulation could represent an additional aid in the study of pathophysiology of psychiatric disorders and in the choice of specific antidepressants in specific clusters of symptoms, especially in comorbidity with internal pathologies. Furthermore limited data, reviewed here, have shown the effectiveness of some antidepressants as pure immunomodulators. However, these considerations are tentative and require experimental confirmation or refutation by future studies.

## INTRODUCTION

In recent years, a growing number of studies are highlighting the links between the immune system and the neuroendocrine system. Neurotransmitters and hormones regulate major immune functions, such as antigen presentation, antibody production, lymphocyte activity, proliferation and traffic, and the secretion of cytokines including the selection of Th1 or Th2 cytokine responses [1]. For several years a role of altered Th1/Th2 cytokine balance in the pathogenesis of various human diseases [2], including psychiatric disorders has been proposed [3]. 

Recently, it was shown that some psychotropic drugs modify the serum concentrations of several cytokines, both in depressed patients and in healthy controls [4], making plausible an immunomodulatory action of these drugs. In fact, the modulation of neurotransmitter systems operated by these molecules seems to affect cytokine production and, consequently, Th1/Th2 balance.

Frequent comorbidities have been reported between psychiatric disorders, such as depression, and several internal pathologies, such as cardiovascular disease [5], cerebrovascular disease [6], inflammatory bowel diseases (depression and Crohn’s disease versus stress factors and ulcerative colitis) [7, 8], psoriasis [9], chronic obstructive pulmonary disease (COPD) [10], rheumatoid arthritis [11], multiple sclerosis [12], systemic lupus erythematosus [13], diabetes [14], thyroid disorders [15], neurodegenerative disorders [16], HCV [17], HIV [18] and cancer [19]. In this regard, data are emerging on both difficulties and potential uses of antidepressants in internistic comorbidities and also as pure immunomodulators. 

A large amount of data has been published on cytokine balances in several human diseases, with still incomplete and sometimes contradictory results. The Th1/Th2 balance, although simplistic from an immunological point of view, represents the basis on which most of the data have been accumulated. Therefore, an overview on this topic is useful to define a reference system in order to frame the interactions between the neuroendocrine system, the immune system and drugs such as antidepressants, which represent the core of interest of this work.

The purpose of this study is to collect available data on the effects of neurotransmitters on cytokine production and compare them with those available on the effects of antidepressants on neurotransmitter and cytokine modulation, providing an overview on the role of Th1/Th2 balance in several chronic diseases, in order to analyze some aspects of the neuro-endocrine-immune (NEI) system that could find a clinical use.

## MATERIALS AND METHODS

A MEDLINE search (1980 - April 2011) was conducted for English-language literature containing combinations of the following key words: “antidepressants”, “SSRI”, “venlafaxine”, “duloxetine”, “bupropion”, “mirtazapine”, “agomelatine”, “monoamine oxidase inhibitors”, “tricyclics”, “norepinephrine”, “serotonin”, “dopamine”, “acetylcholine”, “cortisol”, “melatonin”, “receptors”, “cytokines”, “Th1”, “Th2”, “asthma”, “allergic rhinitis”, “eczema”, “rheumatoid arthritis”, “multiple sclerosis”, “type 1 diabetes mellitus”, “immune thyroid disease”, “Behcet’s disease”, “IgA nephropathy”, “metabolic syndrome”, “essential hypertension”, “dyslipidemia”, “obesity”, “insulin resistance”, “type 2 diabetes mellitus”, “hypercoagulability”, “atherosclerosis”, “cardiovascular diseases”, “acute coronary syndromes”, “acute myocardial infarction”, “chronic heart failure”, “psoriasis”, “chronic obstructive bronchopneumopathy disease”, “Crohn’s disease”, “ulcerative colitis”, “osteoporosis”, “ageing”, “Alzheimer’s disease”, “Parkinson’s disease”, “HIV”, “HCV”, “herpes virus”, “leishmania”, “syphilis”, “tuberculosis”, “cancer”, “metastasis”, “psychiatric disorders”, “mood disorders”, “anxiety disorders”. The references of published articles identified in the initial search process were also examined for any additional studies appropriate for the review.

## NEUROHORMONAL-CYTOKINES INTERACTIONS AND NEI STRESS-ADAPTATION SYSTEM

### Cytokines and Th1/Th2 Balance

Since several years it was proposed that naïve T lymphocytes (Th0) differentiate into subsets of CD4+ T cells that produce distinct types of cytokines [20]. Interleukin (IL)-12, produced by antigen-presenting cells such as monocytes/macrophages and dendritic cells, in concert with IL-18 and Interferon (IFN)-γ promote the differentiation of Th0 cells towards the Th1 phenotype; Th1 cells primarily secrete IFN-γ, IL-2 and Tumor Necrosis Factor (TNF)-β; IL-1, IL-12, TNF-α and IFN-γ (in addition to downregulation of Tumor Growth Factor (TGF)-β) also stimulate the functional activity of cytotoxic cells, natural killer cells and activated macrophages, which are the major components of cell-mediated immunity [1]. Th2 cells produce IL-4, IL-5, IL-6, IL-10 and IL-13 that promote Th2 response stimulating the growth and activation of mast cells (resulting in degranulation and histamine release) and eosinophils (under action of IL-5), the differentiation of B cells into antibody-secreting B cells and the B cell immunoglobulin switching to IgE (under action of IL-4 and IL-13), which are the major components of humoral immunity [1]. These T cells subsets reciprocally regulate themselves since IL-12 and IFN-γ inhibit Th2, while IL-4 and IL-10 inhibit Th1 cell activities: so Th1 and Th2 responses are mutually inhibitory. In this regard, the Th1/Th2 balance hypothesis involves homeostasis between Th1 and Th2 activity that directs different immune response pathways: Th1 cells lead the cell-mediated immunity to fight intracellular pathogens such as viruses and eliminate cancerous cells, while Th2 cells lead the humoral activity to fight extracellular organisms [2]. 

### Neurotransmitters and Cytokines (Table 1)

Evidence accumulated over the last 2-3 decades indicates that neurohormonal messages from the brain superimpose upon and interweave with cytokine balance and immune functions [1].

Cytokine-releasing cells express functional receptors for neurotransmitters, glucocorticoids and cytokines which include the G-linked NE and 5-HT receptors such as β2 [21], 5-HT1A, 5-HT2A, 5-HT1B, 5-HT3 [22-26] and 5-HT transporter [23], the glucocorticoid and mineral corticoid receptors [27], and the five families of cytokine receptors, which are constitutively expressed [28, 29]. NE and 5-HT receptors activate cAMP-dependent pathways which affect both the synthesis and release of cytokines and cellular proliferation [22], while, upon binding cortisol, cytoplasmic/nuclear glucocorticoid receptors undergo dimerization and translocation to the nucleus where they act to modulate lymphocyte proliferation and cytokine gene transcription [30]. 

Recent evidences indicate that both glucocorticoids and catecholamines (NE and epinephrine) systematically mediate a Th2 shift suppressing Th1-cytokine, such as IL-12, IFN-γ, IL-1, TNF-α, and up-regulating Th2-cytokine, such as IL-10, IL-4 and IL-6, production, through stimulation of classic cytoplasmic/nuclear glucocorticoid receptors and β2 adrenergic receptors, respectively [1, 31]. Specifically, since β2 adrenergic receptors are expressed on Th1 cells, but not on Th2 cells [21], NE does not affect directly the cytokine production by Th2 cells; however NE stimulation of β2 adrenergic receptors on Th1 cells suppresses the production of IL-12, the main inducer of Th1 response that stimulates the release of IFN-γ and inhibits that of IL-4; so the NE suppression of IL-12 on Th1 cells indirectly determines the reduction of IFN-γ and the increase of IL-4, thus shifting the Th1/Th2 balance towards Th2 polarization [1]. For instance, since catecholamines up-regulate IL-6 production, the chronic hyper-noradrenergic state may drive the increase in systemic IL-6 levels [1]. Histamine, through activation of H1 and H2 histamine receptors inhibits IL-12, TNF-α and INF-γ, but potentiates IL-10 and IL-6, driving to a Th2 shift [1]. 

On the other hand, 5-HT appears to mediate a Th1 shift. Previous studies have shown that physiological doses of 5-HT (0.15 a 1.5 µg/mL) induce the secretion of the pro-inflammatory cytokines IL-1β, IFN-γ and TNF-α, while at supraphysiological doses (15 µg/mL) the secretion of the same cytokines decreased [32]. Indeed, some evidence shows that 5-HT seems to increase IL-10 and IL-6 and decrease IL-12, by binding to 5-HT3, 5-HT4 and 5-HT7 receptors [33], while other studies demonstrate that 5-HT reduces the formation of antibodies, the humoral arm of the immune response mediated at least in part by the Th2 system, through the group of 5-TH1 receptors [34]. Overall, the increase in serotonergic tone would lead to a reduction in noradrenergic tone [35] and in dopaminergic tone [36], reduction in turn correlated with the increase of the cholinergic one [37, 38]. Some evidences show that dopamine (DA) dose dependently polarize the Th2 differentiation in naive CD4+ T cells *via *D1-like receptors [39], although other studies bring back conflicting data [40, 41]. Stimulation of nicotinic cholinergic receptors appears instead to shift T cells toward Th1 pattern through upregulation of Th1-specific transcription factors [42]. Finally it should be noted that 5-HT is also the precursor of melatonin, which enhances Th1 response increasing the production of IL-2 and INF-γ and decreasing the production of IL-10, *via *nuclear receptor-mediated transcriptional control like the melatonin receptor RZR / ROR [43, 44]; also, one study reported an increase in production of IL-6 by the same melatonin [44]. 

The complex mode of action of neurotransmitters on the Th1/Th2 balance should be analyzed taking into account the molecular context in which each neurotransmitter acts, impact of which also depends on T cells activation state, receptor subtypes expression, neurotransmitter dose and other co-released molecules [45].

### The NEI Stress-adaptation System

From a physiological perspective, environmental stress activates noradrenergic system, hypothalamus-pituitary-adrenal (HPA) axis with cortisol release and Th2 shift, components of the NEI stress-adaptation system [46]. Such systems, in turn, induce a compensatory response with feedback mechanisms in serotonergic system and Th1 components [47]: for example, cortisol downregulates 5-HT1A somatodendritic presynaptic autoreceptors in the raphe nuclei (which play an inhibitory role on 5-HT release), thus enhancing serotonergic tone [47], and, in turn, influencing Th1 response [32]. In adaptive stress (mainly predictable and voluntary), these systems ideally compensate in order to achieve adaptation to higher levels of stress, through epigenetic mechanisms that converge on receptor sensitization/desensitization (such as 5-HT1A), regulation of neurotrophic factor expression (such as BDNF, VEGF and NGF), neurogenesis and synaptic enhancement (due to NMDA receptor modulation and LTP mechanism) on specific neural networks (such as hippocampus network), restoring neurotransmitter and cytokine levels on physiological values (similarly to neuromuscular system adaptation during repeated physical exercise) [47-49]. During inflammation, the activation of the stress system, through induction of a Th2 shift protects the organism from systemic "overshooting" with Th1/pro-inflammatory cytokines [1]. Under certain conditions, however, stress hormones and activation of the corticotropin-releasing hormone (CRH)/substance P - histamine axis may actually facilitate inflammation, through induction of IL-1, IL-6, IL-8, IL-18, TNF-α and CRP production [1]. On the other hand, pro-inflammatory cytokines, such as IL-1, IL-6 and TNF-α, released during immune response and inflammation activate the central components of the stress system, activate the hepatic synthesis of acute phase proteins and alter neurotransmitter networks activity, inducing the “sickness behavior” (fever, sleepiness, fatigue, loss of appetite and decreased libido) [1]. 

## HUMAN CHRONIC DISEASES AND TH1/TH2 BALANCE

For several years a role of altered Th1/Th2 cytokine balance in the pathogenesis of various human diseases has been proposed [2]: a distinct Th1/Th2 divergence determine resistance versus susceptibility to several diseases [50]. 

### Autoimmune and Allergic Diseases

Several autoimmune diseases, such as rheumatoid arthritis, multiple sclerosis, type 1 diabetes mellitus and immune thyroid disease, are characterized by dominant Th1 responses, with an excess of IL-12 and TNF-α production and a deficit of IL-10 production, critical factors in the proliferation and differentiation of Th1-related autoreactive cells [51]. On the other hand, allergic diseases, such as asthma, allergic rhinitis, eczema and IgE-mediated food allergy, are characterized by dominant Th2 responses, with overproduction of IL-4, IL-5, IL-9 and IL-13, resulting in activation of mast cells (and consequent histamine release), eosinophils and shifting to IgE production [52, 53]. 

Multiple sclerosis seems to be associated with a predominantly Th1 cytokine pattern, characterized by increased levels of IFN-γ, TNF-α, IL-12, IL-23 and IL-27 as well as IL-6 and decreased levels of IL-10 and TGF-β [54-56], although other cell subsets such as Th17 cells, regulatory T cells and B cells also appear to be involved [57]. However, this framework could represent the result of a multistep process related to genetic trait, virus infection, activation of the stress system and fluctuations in the Th1-Th2 balance. Stressful life events seem to reduce the clearance of virus infection or permit the episodic reactivation and dissemination of latent viruses (such as herpes virus), probably through overactivation of the HPA axis and the Th2 switch (current multiple sclerosis treatment includes agents for acute relapses, such as corticosteroids, and disease-modifying agents, such as the antiviral IFN-β, which has a beneficial effect on relapsing/remitting multiple sclerosis [58]); patients with multiple sclerosis generate autoreactive T cells that at some points differentiate into Th1 phenotype cells, which are the major players in the late disease maintaining a continuous destructive cell mediated immune response against brain and spinal cord antigens resulting in demyelination [59-61].

In rheumatoid arthritis, Th1 cytokines such as TNF-α, IFN-γ, IL-1, IL-12 and IL-18, as well as IL-23/IL-17 axis, contribute to joint inflammation, cell infiltration and destruction [62-68]; there also appears to be a compensatory anti-inflammatory response in synovial membrane, suggesting a protective role of Th2 cytokines [62]. However, transient Th2 phenotype, characterized by predominance of IL-4 and IL-13 and marked absence of IFN-γ, might be relevant in the early disease [69] and linked to B cell hyperactivity resulting in aberrant autoantibodies (e.g. rheumatoid factor and anti-cyclic-citrullinated antibodies) and immune complexes, that can also be found prior to the clinical onset and may be important in the initiation of the disease [66-68].

Studies on type 1 diabetes have shown that destructive insulitis is mediated through a Th1-dominated autoimmune response associated with an increased expression of IL-1, IL-2, IL-12, TNF-α and IFN-γ [70], as well with unregulated IL-23/IL-17 response [71], and cell mediated beta-cell destruction; this has been suggested by some to arise relatively late in the disease process after a latent period of non-destructive Th2 islet autoimmunity associated with an increased expression of IL-4, IL-10 and TGF-β and with the appearance of autoantibodies targeting, for example, insulin and glutamic acid decarboxylase [72].

About autoimmune thyroid diseases, some studies suggest that Hashimoto thyroiditis is characterized by Th1-dominant response associated with an increased expression of IFN-γ, TNF-α, IL-2, IL-12, IL-18, IL-1β, IL-8 and Th17 system and cell mediated thyroid infiltration and destruction [73-75]. On the other hand, the predominance of Th2 cytokines such as IL-4, IL-5, IL-6 and IL-10 and the related humoral pattern of immune reaction associated with B cell activation and production of autoantibodies against thyroid antigens, characterize Graves disease and early stages of Hashimoto thyroiditis: in Graves disease autoantibodies hyper-stimulate thyroid function, while in Hashimoto thyroiditis, on the contrary, the autoantibodies presence precedes the onset of subclinical and clinical hypothyroidism (thyroid peroxidase antibodies are quantitatively related with the IL-10 levels, which then gradually decrease as the disease progresses) [73-77].

Other diseases characterized by a Th1 polarized response are Behcet’s disease, in which there are increased levels of IFN-γ, IL-12 and IL-13 and reduced levels of IL-4 [78] as well as vitamin D [79], and IgA nephropathy, in which there is an increase in the ratio IFN/IL-10 and increased levels of IFN-γ, TNF, IL-1, IL-2 and IL-8 [80, 81]. 

Interestingly, some studies show that a hypoactive stress system may facilitate or sustain the Th1 shift in Th1-mediated diseases, such as rheumatoid arthritis [82-85].

On the other hand, systemic lupus erythematosus seems to be an autoimmune disease characterized by dominant Th2 response; however, during advanced stages of disease there is substantial ongoing Th1 cell-mediated inflammation. An important genetic load and environmental factors are involved in the pathophysiology of the disease, such as sunlight exposure and abnormal estrogen metabolism or environmental estrogen exposure (in postmenopause or in contraception use), whereas there is little evidence of infection; notably, estrogens, as well as sunlight, seem to shift the cytokine balance towards Th2 response, increasing IL-4, IL,10 and TGF-β and decreasing IFN-γ, TNF-α and IL-12, whereas androgens seem to enhance the Th1 response [86, 87]: the shifting to Th2 response, characterized by the increase of IL-10 and IL-6 and the decrease of IL-2 and IL-12, together with defective immune regulatory mechanisms such as the clearance of apoptotic cells and immune complexes, leads to B cell hyperactivity and production of pathogenic autoantibodies [86]. During advanced stages of disease there is substantial ongoing Th1 (and possibly Th17) cell-mediated inflammation, such as in lupus nephritis, characterized by increase in IFN-γ, TNF-α, IL-12, IL-17, IL-23 and IL-18 [88-93], suggesting diversified immuno-modulatory interventions depending on the stage of disease [88].

Regarding allergic diseases, although the onset of acute atopic dermatitis is strongly associated with the production of Th2 cytokines, notably IL-4, IL-5, IL-13 and IL-31, the expression of IFN-γ and IL-17 rather than IL-4 predominates in older lesions, indicating a cytokine switch from Th2 in the acute phase towards Th1 in the chronic phase [94].

Also, allergic asthma seems to be mediated by a Th2-dominated response, related to IL-4, IL-5, IL-9 and IL-13 increase [95-96]; however, after migration to asthmatic lung, cells switch on Th1 cytokines such as IFN-γ and TNF-α and, together with tissue-infiltrating Th17 cells, may modulate the local chronic inflammatory response [97-99]; moreover, Th2 cells are recruited efficiently to sites of tissue inflammation, creating a vicious circle in association with disease exacerbations related for example to bacterial or viral respiratory tract infections [100]. 

In summary, several immune-mediated diseases seem to be characterized by a multistep process resulting from complex interaction between predisposing genetic traits, infections, episodic and chronic activation of the neuroendocrine stress system and fluctuations in the Th1-Th2 system. The changes in cytokines expression show often a mixed pattern. Nevertheless, in the early stages Th2 activation is often detected, correlated with humoral response and allergic reactions or production of autoantibodies, which in some cases appear clinically silent, while in other determine clinically significant pathophysiological phenomena. In the late stages the immune response seems to show in several diseases a switch from Th2 to Th1 correlated to cell-mediated organ inflammation. Episodic or chronic activation of the neuroendocrine stress system due to several causes, including stressful events and intercurrent infections together with genetic polymorphisms and epigenetic factors, affects the Th2 response, while the Th2-to-Th1 switch has been in some cases linked to a hypoactive stress system.

### Inflammatory, Metabolic and Cardiovascular Diseases

Chronic activation of the Th2-related stress system seems to lead to a Th1 switch with elevation of both Th1 and Th2 inflammatory cytokines that result in chronic systemic inflammation associated with a cluster of metabolic disturbances named metabolic syndrome, including arterial hypertension, dyslipidemia and obesity (specifically the visceral type), insulin resistance and/or diabetes type 2, in addition to endothelial inflammation and hypercoagulability of the blood [101]. These immune and metabolic changes increase all cause mortality, primarily cardiovascular due to atherosclerosis, and also cancer and infection-related [1, 102].

Essential hypertension seems to be associated with a cytokine pattern biased towards the Th2 system, with an increase of IL-4, IL-7, IL-13 [103], IL-6 [104] and TGF-β [105], as well as the pro-inflammatory cytokine TNF-α [104]. This pattern could be related to the hyper-reactivity of the stress system found in patients with essential hypertension. Mental stress induces phenylethanolamine N-methyltransferase, which may act as a DNA methylase, causing the NET gene silencing, which in turn, may exaggerate autonomic responsiveness [106]: this epigenetic modification has been hypothesized to underlie both essential hypertension and panic disorder, conditions frequently associated to each other [107]; moreover, cortisol responses to mental stress are greater and more persistent in people at high risk for hypertension relative to low-risk normotensives [108, 109].

Dyslipidemia is associated with an impaired Th1 and enhanced Th2 responses [110]. While an increase of IFN-γ was found in course of moderate hypercholesterolemia, severe hypercholesterolemia seems to be characterized by a Th2 shift, with an increase of IL-4 [111]: notably, cortisol and catecholamines, released during stress response, are involved in fat mobilization [112, 113]. 

Obesity, especially the visceral type, is associated with a predominantly Th1 cytokine pattern, with increased levels of IFN-γ, TNF-α, IL-1 and IL-8, as well as of the Th2 proinflammatory cytokine IL-6 [114-118]; furthermore leptin, an adipocyte-secreted hormone, modulates Th cells toward a Th1 phenotype [118]. However, it was shown in an animal model that stress exposure during the adolescent period decreases subcutaneous fat content, without change in visceral fat, and consequently increases the visceral fat / subcutaneous fat ratio in adulthood; treatment with SSRIs during stress exposure prevented later effects on body fat distribution [119]. It is possible that, while Th2-related acute stress reduces the subcutaneous fat to the mobilization of energy reserves, chronic stress, in association with a chronic activation of the HPA axis and a Th2-to-Th1 switch, facilitates Th1 inflammation and visceral fat distribution. HPA axis activation increases cortisol production; Cushing’s syndrome is the best evidence showing a link between chronic hypercortisolemia and accumulation of central fat; since fat cells catabolize glucocorticoids, hypertrophy of visceral fat could represent, among others, an adaptive response to chronic hyperactivity of the HPA axis, and so an adaptive role in response to stress; in fact abdominal obesity is associated with an increased cortisol clearance [120, 121].

Insulin resistance seems to be associated with a predominantly Th1 cytokine pattern: TNF-α (and to a lesser extent IL-1 and IL-6), produced by visceral fat among others, inhibits insulin signaling pathways, leading to insulin resistance [116, 122-124]. Type 2 diabetes is associated with an elevation of Th1 subset, with an increase of IFN-γ and IL-17 [125], while insulin induces a shift in T cell differentiation towards a Th2 response, decreasing IFN-γ and increasing IL-4 [126]. Resulting hyperglycemia determines, besides other effects, a decrease of LDL clearance and endothelial dysfunction, implicated in the pathogenesis of atherosclerosis [116, 122]. 

Atherosclerosis seems to be a chronic inflammatory disease of vascular endothelium related to a prevalent Th1 cytokine profile, in most cases initiated by chronic hypercholesterolemia, with an increase of Th1 cytokines with atherogenic activity such as IFN-γ, TNF-α, IL-1β, IL-8, IL-12 and IL-18 and a decrease of Th2 cytokines with anti-atherogenic properties such as IL-4, IL-5, IL-10 and IL-13 [127-130], while the increase of systemic mediators such as IL-6 appears to lead to the increased tendency of thrombosis [131]. The Th2-related dyslipidemia appears to be associated in chronic with the increase in oxidized LDL, especially in the presence of hyperglycemia and smoking (in addition probably to other factors such as infections with chlamydia pneumoniae and/or human cytomegalovirus [131]), resulting in the activation of macrophages and T cells that release predominantly Th1 cytokines, and so lead to chronic inflammation of the vessel wall and plaque formation [127-129]. 

In this regard it is notable that TNF augments inflammation, TNF and IFN-γ induce coagulation, IL-1β induces coagulation and fibrinolysis, IL-8 augments synergistic inflammation and coagulation, IL-6 augments coagulation and inhibits fibrinolysis, IL-10 inhibits inflammation and fibrinolysis and IL-4, IL-13 and TGF-β act for anticoagulation [132]: so hypercoagulability of the blood is associated with an inflammatory state [133] characterized by Th1 predominant response, not only present in atherosclerosis, but also in visceral obesity [134], disseminated intravascular coagulation [135] and cancer [136].

Atherosclerosis represents one of the key factors of cardiovascular and cerebrovascular diseases: the inflammation-related predominant Th1 response has also been implicated in the pathophysiology of chronic heart failure [137, 138] and heart failure after acute myocardial infarction [139]. The stress-related Th2 predominant response seems to be associated with acute coronary syndromes: acute exogenous or endogenous stress factors can trigger unstable angina, sudden cardiac death and acute myocardial infarction [140, 141], condition in which was found, in relation to sympathetic system activation and catecholamines release, an increase of IL-6 [142, 143] and IL-10 [144, 145]. Th2 polarized response also promotes fibrosis in general, and cardiac fibrosis in particular [146]. Regarding the cytokine imbalance in cardiovascular disease it should be noticed that atorvastatin can regulate the polarization of Th1/Th2 through the inhibition of Th1 cytokines [138, 139], while beta-blockers and angiotensin-converting enzyme inhibitors can decrease serum levels of IL-6, which correlates with NYHA class in chronic heart failure [147]. Levels of IL-6 in the serum of acute stroke patients have been reported to also correlate with stroke severity; serum concentrations of IL-6, IL-8 and IL-10 were higher in asphyxiated neonates; while cerebral ischemia initiates a Th1-prevalent inflammatory response associated with induction of a variety of cytokines, including TNF-α, IL-1β, IL-8, IL-18 and IL-6 [148-151].

Mixed Th1/Th2 pattern, related to chronic hyperactivation of proinflammatory cytokines mainly belonging to the Th1 group, seems to be associated to the pathophysiology and evolution of a number of other conditions. Several inflammatory diseases show a prevalence of Th1 response, such as psoriasis, in which there are high levels of IL-2 and IFN-γ and low levels of IL-4 and IL-10 [152, 153]. In COPD, the increase of the Th1 cytokines IFN-γ, TNF-α, IL-2 and IL-8 and the decrease of the Th2 cytokines IL-4 and TGF-β seem to be associated with progression to advanced stages of the disease [154, 155]. Furthermore, alterations of Th1/Th2 balance also appear to be relevant in the difference in clinical and pathophysiological presentation of diseases such as inflammatory bowel disease: the prevalence of a Th1 response involving overproduction of IL-12, IFN-γ and TNF-α manifests in the form of Crohn’s disease, while the prevalence of a Th2 response probably driven by overproduction of IL-13 manifests in the ulcerative colitis [156]. Osteoporosis is associated with low-grade inflammation both Th1 than Th2 type, since the bone turnover is regulated in a complex way: TNF-α, IL-1, IL-6, IL-11, IL-17 and glucocorticoids enhance bone resorption, while IFN-γ, IL-12, IL-18 and IL-4 inhibit bone resorption; osteoporosis is in fact associated both with diseases with a predominant Th1 cytokine pattern, such as atherosclerosis and rheumatoid arthritis, and conditions with a Th2-dominated cytokine pattern, such as ageing, menopause, pregnancy, HIV, steroid or cyclosporine therapy [157].

Other physiological and paraphysiological conditions are associated with changes in cytokine balance potentially favoring different types of diseases: ageing, pregnancy and menopause that have a Th2-dominated cytokine balance. During reproductive adulthood there is a relative bias towards Th1 immunity, which reverses to Th2 dominance, together with an increase of pro-inflammatory cytokines such as TNF-α, IL-1 and IL-6, during post-reproductive senescence, with impaired cellular response and increased susceptibility to viruses, intracellular pathogens and neoplastic diseases [158, 159]; during pregnancy a Th2 shift might be favored by the increase of cortisol and endorphins releases [160].

### Neuroinflammation

Neuroinflammation appears to be central in degenerative diseases such as Parkinson disease and Alzheimer disease. Cytokines are expressed physiologically in CNS cells and are important for the development and function of the brain [58]. Furthermore, increasing evidence suggests that changes in cytokines levels outside the brain cause changes in cytokine expression and activity in the brain, and vice versa [161]. In the brain, activated glia, such as microglia and astrocytes, are the major cell types that participate in the inflammatory system both as sources and targets of cytokines [162]; the activation of these cellular systems seems to promote apoptotic cell death and subsequent phagocytosis of DA neurons in Parkinson disease or to be associated with amyloid plaques formation and neurodegeneration of hippocampus and other brain areas in Alzheimer disease [162-164]. In parkinsonian patients, increased levels of TNF-α, IFN-γ, IL-1β, IL-2, IL-4, IL-6, IL-10 and TGF concentration in serum, cerebrospinal fluid and nigrostriatal DA regions and decreased ones of neurotrophins were found [163-167]. Yet, Th1 cytokines such as IFN-γ seem to be implicate in apoptotic neuro-degeneration [168-170], while Th2 cytokines such as IL-10 seem to protect against inflammation-mediated degeneration of dopaminergic neurons in the substantia nigra [171, 172], possibly representing a compensatory increase. Furthermore, while damage of DA neurons in the nigrostriatal system seems to be associated with an exacerbated pro-inflammatory response [173], levodopa treatment correlates with reduction of IL-2 production [174]. In Alzheimer disease patients, increased levels of Th1 cytokines such as IFN-γ, and decreased levels of Th2 cytokines such as IL-4 have been found [175]. In a study on dementia-free subjects high levels of IL-1β and TNF-α were associated with an increased risk of developing Alzheimer’s disease [58], while the induction of Th2-biased responses seems to improve the clinical and pathophysiological condition in an Alzheimer’s mouse model, increasing IL-10 and reducing IFN-γ, TNF-α, IL-2 and IL-4 [176, 177]. Yet also Th2 cytokines such as IL-6 seem to have a pathogenic role in neurodegeneration [58, 178]; in this respect it is notable that brain NE levels, which potentially enhances Th2 response, may be elevated at least in some cases or in some phases of Alzheimer disease, considering a direct evidence of elevation in NE or in its metabolites in Alzheimer’s disease and the frequent comorbidity with bipolar disorder or hypertension, disorders in which elevated NE may be involved [179].

In summary, neuroinflammatory pathologies seem to be polarized to Th1 response, although Th2 component appears to play a compensatory role or to influence some stages of disease.

### Chronic Infections and Cancer

The Th1-to-Th2 switch hypothesis has been proposed for the first time in chronic infections like HIV, in which it is highlighted that Th1 hyperactivation would result in a resistance or infection control, while the decline in Th1 cell activity and the increase in Th2 cell activity would result in a progression of the infection [180]. It was shown that environmental or internal stressors play a role in HIV disease progression, affecting immunity (CD4 cell counts) and viral set-point (plasma viral load) both in simian [181, 182] and in human [183, 184], and this could be correlated with a Th1-to-Th2 switch dependent on catecholaminergic and HPA axis activation [185-187]; moreover, the Th1-to-Th2 switch could be indicative of an allergic response to viral proteins [188] or other factors. Nevertheless, it was proposed that Th2 cytokine inhibitors and immune response modifiers could lead to a Th2-to-Th1 reversion, inducing a revival of the anti-viral Th1 response [188]. In other chronic systemic infections, a disease control due to Th1 hyperactivation and a disease progression due to Th2 switch were also observed [189], such as in HCV [190-192], herpes virus [193] (the use of topical immunomodulators which stimulate the release of Th1 type cytokines against HSV infected cells, as well as against human papillomavirus and cancerous skin lesions has been proposed [194, 195]), leishmania [196], syphilis [197] and tuberculosis [198-200] (in which HPA axis activation causes increased cortisol secretion possibly resulting in Th2 shift [201]), as well as in atopic diseases such as atopic eczema (in which a dominant Th2 response would replace the Th1/Th2 mixed pattern during the evolution of eczematous lesions) [1].

Regarding cancer, a Th1 phenotype appears to exert anticancer effects [202, 203], although the Th1 cytokine effects may be bimodal, since the inflammatory nature of the Th1 response may allow the development of DNA damage that facilitates transformation [204]. On the other hand, the progression from primary tumor to the metastatic phenotype could correlate to a Th2 switch [205], since it was found, with the passage of time, an increase of Th2 cytokines such as IL-4, IL-6, IL-10 and TGF-β and a decrease of Th1 cytokines such as TNF, IFN-γ and IL-1 in several metastatic cancers that became more evident during disease progression, such as hepatocellular carcinoma [206, 207], head and neck squamous cell carcinoma [208, 209], hypopharyngeal carcinoma [210], esophageal squamous cell carcinoma [211], pulmonary adenocarcinoma [212], breast cancer [213], melanoma [214], lymphoma [215, 216] and pancreatic carcinoma [217-219]. The Th1-to-Th2 switch mechanism could be influenced either by systemic factors such as environmental events that activate the stress system [220] or by local factors such as hypoxia [221]. Similarly to what was observed about the progression of HIV in stress conditions, it was demonstrated that the persistent activation of sympathetic and HPA systems in chronic stress response impaired the immune surveillance of tumors, resulting in the progression of some types of cancer, in association with a shift in the balance of Th1-Th2 immune response [220, 222], such as in breast cancer [223]. In this regard, the connection between the neurometabolic and immune systems appears to be relevant. It has been shown that the indoleamine 2,3-dioxygenase (IDO) enzyme, involved in the catabolic pathway of tryptophan (precursor of 5-HT), is overexpressed both in tumor cell and antigen-presenting cell in tumor draying lymph node, where it promotes the establishment of peripheral immune tolerance to tumor antigens [224]. Therefore, in course of chronic immune activation, decreased serum tryptophan, associated with 5-HT reduced synthesis [225, 226] and hypercortisolism [226], was found parallel to the course of malignant diseases [227]. These alterations appear to be associated, on one hand, with generation of immune tolerance in patients with advanced cancer (facilitating the survival and growth of tumor cell expressing unique antigens that would be recognized normally as foreign by T cells) [224, 225, 227], on the other, with irritability, lack of impulse control and mood disorders [225-227]. Some experimental approaches with IDO inhibitors are underway as a new immunomodulatory treatment [224, 228]. Interestingly, HIV infection is also associated with increased tryptophan catabolism by the IDO, contributing to perpetuation of HIV infection into its chronic phase by dampening efficient immune anti-viral responses [229]. Similarly, an influence of tryptophan degradation on different aspects of autoimmune disorders such as multiple sclerosis [230], rheumatoid arthritis [231] or systemic lupus erythematosus was detected [232]. A compensatory increase in IDO activity in models of rheumatoid arthritis was shown, indeed systemic inhibition of tryptophan catabolism correlated early with an increased production of IFN-γ and IL-17 and later with an increased infiltration of Th1 and Th17 cells in the inflamed joints, increasing disease severity [231]. Furthermore, it was noted that high IDO activity in winter predicted subsequent activation of systemic lupus erythematosus in spring and summer [232]. On the other hand there is evidence that low tyrosine and phenylalanine diet limit tumor growth in animal models, in relation to the reduction in blood levels of these amino acids involved in the catecholamine synthetic pathway; however, this diet has not found clinical applicability in the treatment of patients with advanced cancer for tolerability issues [233]. 

So, insufficient immunosurveillance is an important aspect in early tumorigenesis and in the pathogenesis of malignant disease; in the later course of cancer, the development of immunodeficiency is considered to be the major reason for disease progression and death [227], and mood symptoms could indicate an immunological failure associated with tumor progression [225-227]. 

In some cases specific therapeutic actions seem to reverse such changes to a Th1 response, increasing Th1 cytokines and decreasing Th2 cytokines, such as operations [211] and chemotherapy [234], but theoretically it is possible that immunomodulation actions on the stress system could enhance the Th1/Th2 balance restore.

In summary, the Th1-Th2 switch paradigm, in which perturbations of the system allow to switch from Th1 to Th2 response or vice versa, can influence the pathophysiology and evolution of several diseases [235]. Environmental or internal stress factors occurring on Th1 chronic activation could induce a Th2 switch which would lead to a progression of the underlying pathology for relative displacement of the immune reserve, in relation to interaction between fluctuations of Th1/Th2 balance and neuro-hormonal balance.

### Psychiatric Diseases

In recent years, a growing body of data was collected on the presence of hormonal and cytokine changes also in various psychiatric disorders, particularly mood and anxiety disorders [3]. In Major Depressive Disorder (MDD), neuroendocrine-immune interactions, tightly regulated by HPA axis under homeostatic conditions [236], cause imbalances in levels of neurotransmitters such as 5-HT and NE, hormones such as cortisol, and also in cytokines such as TNFα and IL-6 (variations of which show the closest association with depression), and also IL-1β, IL-4, IL-2, IL-8, IL-10 and IFN-γ, that contribute to the behavioral and immune disturbances observed in these patients [237-245]. MDD seems to be associated to HPA axis hyperactivity [241], as confirmed by hypercortisolism, one of the most consistent clinical features of MDD [246, 247], and desensitization of glucocorticoid receptor, the most important regulator of HPA axis negative feedback system [248] (variations in cortisol levels are directly related to the activation index of HPA axis [249]). Previous studies reported that MDD patients without pharmacological treatment present polarization towards a Th2 type circulatory cytokine profile [250-252] and other alterations in the immune response such as changes in antibody levels and complement deficiencies [253], numeric alterations in several sub-sets of lymphocytes [254] and increase in the development of some infectious and tumorous conditions [255] in non-treated patients. This could be explained by the fact that hypercortisolism and NE systematically mediate a Th2 shift [1, 31, 250]. HPA axis hyperactivity could lead in some cases to deficiency in 5-HT compensatory tone. In fact, in animal models of depression cortisol has been shown to negatively regulate the 5-HT1A receptor in a dose-dependent manner [256], and other research has shown in lymphocytes from MDD patients a decrease in the density of the 5-HT transporter [23] and inhibition of 5-HT1A receptor function [22]. So, chronic excess in cortisol levels, index of HPA axis hyperactivity, downregulating 5-HT1A postsynaptic receptors in amygdala and cortex (which play an inhibitory role on glutamatergic tone), could lead to amygdala glutamatergic hyperactivity (and excitotoxicity) due to decrease in 5-HT activity, altering dendrites and spine number in such neurons network [47, 257]; 5-HT activity deficiency, in turn, might be related to lower levels of Th1 cytokine with Th2 shift and to the onset of anxiety and depressive symptoms [258]. 

Similar cytokine findings have been evidenced also in the course of anxiety disorders: in obsessive compulsive disorder higher levels of IL-6 and TNF-α were found, while in posttraumatic stress disorder, in addition to IL-6 and TNF-α increasing, also low cortisol and high DHEA levels were found [259-261]; furthermore, a systematic shift in cytokine balance towards a Th2 profile has been suggested in the Gulf War syndrome [262]. The alteration in cytokine patterns does not seem to be much different in anxious and depressed patients, possibly reflecting a substantial psychopathological overlap or the frequent comorbidity of anxiety and depressive symptoms in the same patient, making it difficult to distinguish homogeneous samples structured on the distinction of individual psychopathological dimensions [263]. In addition, there may be other confounding factors that make it difficult to relate specific sets of symptoms with specific patterns of cytokine alterations. For example, the frequent comorbidity of cigarette smoking with mood disorders [264] may contaminate the cytokine pattern related to HPA axis hyperactivity [1], often detected in the course of mood disorders [265], pursuant to a stimulation of Th1-type cytokines due to chronic activation of nicotinic receptors by nicotine in cigarettes [42]. Another confounding factor could be represented by body weight, since adipose tissue secretes TNF-α and IL-6 so that plasma levels of these cytokines are proportional to body mass index and are further elevated in patients with visceral obesity [266].

So, depressive and anxiety syndromes seem to be mainly characterized by chronic hyposerotonergic state, HPA axis hyperactivation and Th2 shift. Nevertheless, other studies suggest an imbalance Th1/Th2 shifted towards Th1 in depression [267, 268]. In this regard, a dimensional approach could lead to further insight of the issue. In melancholic depression, condition in which patients have anxiety, insomnia, anorexia and circadian variation with worsening in the morning, appears to be associated with significantly higher CSF NE and plasma cortisol levels that are increased around the clock, with inappropriately high plasma ACTH and CSF CRH levels considering the degree of their hypercortisolism. These data suggest a central hyper-noradrenergic state in association to hyperfunction of central CRH pathway [269]; furthermore, the chronic hyper-noradrenergic state may drive the increase in systemic IL-6 levels, since NE up-regulates IL-6 production, and, theoretically, to a Th2 shift [1]. On the other hand, atypical depression, condition in which patients have hypersomnia, hyperphagia and fatigue, appears to be associated with a central hypo-noradrenergic state in association to hypofunction of central CRH pathway [270, 271] and so, hypothetically, to a Th1 shift. It is noteworthy that atypical depression, ideally characterized by Th1 shift, seems to belong to bipolar depression [272]. On the other hand, during mania, IL-2, IL-4, IL-6 and IL-10 were found to be increased while IFN-γ decreased, ideally reflecting a Th2 shift [273-275]. Finally, both childhood and adult schizophrenia have been shown to be accompanied by elevated expression of TNF-α, IL-1 and IL-6 [276, 277]. 

In summary, unipolar depressive disorders and anxiety disorders might be intended as pathologies of NEI stress-adaptation system, mainly characterized by functional alterations and neuronal remodeling of anterior cingulate cortex, amygdala, hippocampus and HPA axis manifested in chronic hypernoradrenergic/hyposerotonergic state, HPA axis hyperactivation and Th2 shift or, in some case, Th1 switch [245, 250, 251, 257, 278]. On the other hand, bipolar depression might be included in primitive affective disorders of cortico-limbic systems (cingulate cortex, striatum, putamen, thalamus, prefrontal cortex and amygdala) [279-281], which in turn could secondarily involve the NEI stress system, since bipolar and anxiety disorders have both been associated with abnormality in the amygdala, one of the main nuclei involved in the regulation of HPA axis [282, 283]. It is possible that the dimensional approach in psychiatric diagnoses, together with attention to various confounding factors, could create homogeneous groups of patients that match the underlying neuroendocrine-immune alterations, to which the models of imbalance and dynamic switching of Th1/Th2 system, already used in other human diseases, could be applied.

## ANTIDEPRESSANTS AND TH1/TH2 BALANCE 

### Antidepressants, Neurotransmission and Cytokines (Table 2) 

Some antidepressants would occur affecting NEI system, acting on neurotransmitter balance (especially on the 5-HT/NE balance) and levels of expression and sensitivity of individual receptor subtypes, which in turn affect the cytokine production and the relative Th1/Th2 balance.

### Selective Serotonin Reuptake Inhibitors (*SSRIs)*

In rigorous and long term clinical study, SSRIs seem to increase Th1 cytokines, such as IL-1β, IL-2 and IFN-γ, and decrease Th2 cytokines, such as IL-4, IL-10 and IL-13, and cortisol levels after 52 weeks treatment in depressed patients [252], although other *in vitro* and short term *ex-vivo* studies reported conflicting results, showing decrease in IL-1β, IL-6, IL-10, IFN-γ and TNF-α after SSRI treatment in a dose dependent manner [284-288]. In that study, administration of SSRI in MDD patients, confirming baseline high levels of cortisol, IL-4, IL-13 and IL-10 (Th2) compared with healthy volunteers, induced clinical remission at week 20 of treatment, concomitantly with an increase in IL-2 and IL-1β levels (Th1) without changes in cortisol level. At week 52 of treatment, SSRI administration induced an increase in IL-1β and IFN-γ levels (Th1), together with a reduction in IL-4, IL-13 and IL-10 levels (Th2) and in cortisol levels (a 30% diminution compared to baseline) [252]. Variations in these parameters could be caused by SSRI effects both on 5-HT and glucocorticoid receptors, as a result of chronic intake of these drugs. SSRIs exert a relatively selective blockade of 5-HT transporter [289], progressively increasing 5-HT levels, also in the circulation [290, 291], and influencing the immune response in a dose-dependent manner [252]. As a consequence, long-term SSRI treatment desensitizes the inhibitory somatodendritic 5-HT1A autoreceptors in the dorsal and medial raphe, and 5-HT neurotransmission is enhanced [292-294]. Furthermore, a desensitization of 5-HT2A and 5-HT2C receptors occurs as a consequence of prolonged exposure to elevate levels of 5-HT [295, 296]. Finally, since 5-HT neurons exert a tonic inhibitory effect on locus coeruleus neurons, it appears that enhancing 5-HT neurotransmission by sustained SSRI administration leads to a reduction in the firing rate of noradrenergic neurons [35]. Thus, drug-mediated enhancement of 5-HT activity exerts immunostimulatory effects on Th1 cytokines [32], possibly acting on 5-HT1A receptors, and concomitant immunoinhibitory effects on Th2 cytokines. Furthermore, it has been proposed that long term SSRI treatment in depressed patients causes a decrease in circulating cortisol levels by reestablishing the down-regulated glucocorticoid receptor sensitivity [27], thus restoring negative feedback by cortisol on the HPA axis [297-299]. Finally it was shown that paroxetine attenuated cyclooxygenase (COX)-2 expression in human T cells [300], considering that COX inhibition due to NSAIDs results in augmentation of the Th1 response by limiting prostanoid synthesis [301].

### Serotonin Norepinephrine Reuptake Inhibitors (SNRIs)

Venlafaxine, a SNRI, appears to have a more complex action on cytokine levels [302]. In several clinical and preclinical studies it was observed that venlafaxine reduces blood levels of IL-12, TNF-α, IFN-γ and increases those of IL-10 and TGF-β1 [303-306]. However, for discussion purpose, it is important to emphasize the dose-dependent effects of venlafaxine on cytokines such as IL-6, a molecule involved in the acute phase response and in the control of Th1/Th2 differentiation towards a Th2 polarization [307]: at low dose venlafaxine appears to reduce serum levels of IL-6 [305, 308], while at higher dose it seems to rather increase levels of IL-6 [309]. These data could be related to the peculiar pharmacodynamics of venlafaxine: the effects on neurotransmission and receptors expression do not seem to differ much from those of SSRIs, at least at low dose [310-312]; nevertheless at higher dose venlafaxine acts as a real SNRI: while at low dose the molecule mainly blocks the reuptake of 5-HT, at high dose the molecule blocks the reuptake of 5-HT and NE to the same extent [313]. Duloxetine, another SNRI, in contrast to venlafaxine has a greater affinity for the NE transporter, blocking to the same extent the reuptake of NE and 5-HT at standard dose [314]. Consequently, chronic administration of this molecule leads, in addition to the desensitization of dorsal raphe nucleus somatodendritic 5-HT1A autoreceptors with increased serotonergic firing [315, 316], also an increase in NE release in frontal cortex and hippocampus due to the desensitization of α2-adrenergic terminal autoreceptor [315, 317]. Our preliminary evidence has shown an increase in the serum levels of IL-6 associated with 6 weeks duloxetine treatment in depressed patients; if the increased levels of IL-6 by duloxetine were confirmed on larger samples, it could be assumed that at the basis of changes in IL-6 serum levels, considering them as a marker of Th1/Th2 balance, operates a 5-HT/NE balance [318]. 

### Norepinephrine Reuptake Inhibitors (NRIs)

To date there are no clinical studies on the interaction between the NRIs reboxetine and atomoxetine and cytokine production. In preclinical studies, treatment with reboxetine seems to enhance IL-10 expression [319], as well as IL1β and its negative regulators, IL-1 receptor antagonist and IL-1 type II receptor, in rat cortex [320]. In a study it was observed that pretreatment with reboxetine inhibited the increase of IL-6 by exposure to IFN-γ in murine cells [321]. In another work, treatment with atomoxetine reduced rat cortical gene expression of IL-1β and TNF-α [322]. Overall, the limited available data could suggest that this class of molecules may shift the Th1/Th2 balance towards Th2 response. It is noteworthy that despite the NRIs selectively block NE reuptake, chronic administration of these molecules causes profound changes in neurotransmission of several systems. Inhibition of brain NE reuptake by acute reboxetine administration results in an inhibition of central noradrenergic activity *via *local increase in NE concentration at inhibitory α2 autoreceptors [323]; however, the sustained elevation of NE synaptic availability due to long term treatment results in desensitization and downregulation of α2 terminal receptor at least at level of prefrontal cortex and dorsal hippocampus, in turn strengthening NE transmission in these areas [324] and attenuating the inhibitory restraint on sympathetic outflow, obtaining an opposite result on noradrenergic tone in relation to time of action and receptor sensitivity [323]. In addition repeated administration of NRIs results in hypersensitization of α1 adrenergic receptor [325, 326] and desensitization of β1 adrenergic receptor [327, 328]. NRIs induce an increase in burst firing of DA cell in ventral tegmental area [329] and selectively increase DA availability in medial prefrontal cortex [329, 330], since in such area NE transporter even reuptakes DA. The desensitization of α2 adrenergic heteroreceptors would increase tonic activation of postsynaptic 5-HT1A receptors [331]. Finally, NRIs seem to have a complex action on acetylcholine (ACh), inhibiting the nicotinic receptors function [332] and also increasing cortical and hippocampal ACh [333], and appear to stimulate the secretion of cortisol (as well as adrenocorticotropin, growth hormone and prolactin) [334, 335].

### Norepinephrine Dopamine Reuptake Inhibitors (NDRIs)

Some evidences showed that bupropion, a NDRI, reduces blood levels of TNF-α, IFN-γ, IL-1β and increases those of IL-10 (effect largely abrogated by β-adrenergic or D1 receptor antagonists and not by a D2 antagonist) [336] and IL-8 [337], shifting the Th1/Th2 balance towards Th2 polarization. Bupropion with its metabolites inhibits reuptake for human transporters for both DA and NE, with slightly greater functional potency at DA transporter than at NE transporter, increasing DA and NE concentrations in prefrontal cortex and DA concentrations in nucleus accumbens [338]. Inhibition of 5-HT reuptake *via *the 5-HT transporter was negligible even at the highest concentration tested [338]. Bupropion and its metabolites do not have appreciable affinity for postsynaptic receptors including histamine, α- or β-adrenergic, 5-HT or DA receptors [338, 339]. Most recent evidence showed that this molecule behaves as a noncompetitive antagonist of several nicotinic ACh receptors [340, 341].

### Other Classes of Antidepressants 

Other classes of antidepressants modulate neuro-transmission in more complex ways than selective reuptake inhibitors and their relatively homogeneous effects on specific neurotransmitters, making it even more difficult to examine the relationship between neurotransmitter modulation and Th1/Th2 balance.

Among these, mirtazapine seems to alter plasma concentrations of several cytokines, increasing levels of TNF-α [342] and reducing those of IL-6 [343] on one hand, inhibiting the production of IFN-γ and increasing the production of IL-4 [343] on the other. Mirtazapine blocks α2 adrenoceptors on noradrenergic neurons (autoreceptors) and on serotonergic neurons (heteroreceptors), 5-HT2 and 5-HT3 receptors and H1 receptor [344]. Long term administration of mirtazapine desensitizes terminal α2 adrenergic hetero-receptor, but not terminal α2 adrenergic autoreceptor [345], leading to an increase in the firing rate of 5-HT neurons (75%) and in the firing rate of NE neurons (30%) [346]. Since mirtazapine blocks 5-HT2 and 5-HT3 receptors, only 5-HT1-mediated transmission is enhanced [347] (the 5HT1A receptor stimulation seems to be directly related to the increase of TNF [348]) and, on the other hand, there is a reinforcement of DA and NE tone in prefrontal cortex [349]. It should also be noted that mirtazapine increases melatonin and decreases cortisol levels [350], effects that overall could account for a shift in balance towards the Th1 response. 

Trazodone, a molecule that blocks 5HT2(A/C), H1 and α1 receptors and also 5-HT transporter, but only at high dose [351], seems to reduce levels of TNF-α, IL-1β [352] and IFN-γ as well [353].

Agomelatine, a novel melatonergic MT1 and MT2 receptors agonist and a 5-HT2C receptor antagonist [354], seems to stimulate IL-2 production, like melatonin, *via *MT1 receptor agonism in human lymphocytes [355]; chronic administration modulates chronobiological rhythms, including temporal organization of cortisol secretion [356] (MT and 5-HT2C receptors exhibit a circadian rhythms of expression), and desensitizes 5-HT2C receptor, which results in NE and DA enhancing only in frontal cortex [354], without affecting sensitization of 5-HT1A receptors [357] or that of suprachiasmatic nucleus neuronal responses [358].

Some studies on tricyclic drugs show that this class of molecules inhibits the secretion of IL-1β, IL-2, TNF-α, IFN-γ [284, 359] and stimulates the production of IL-10 [360]. Also, one clinical study reports an increase of IL-6 following tricyclic administration [309], while other preclinical studies do report a reduction [359, 361, 362] and still others show no change [363, 364]. Though overall tricyclics mainly block NE and, to a lesser extent, 5HT reuptake, the effects of individual molecules within this class of antidepressants are heterogeneous, enhancing noradrenergic and serotonergic transmission within a different spectrum, as well as each molecule blocks with different affinities M1, α1 and H1 receptors [365], also potentially involved in immuno-modulation. This makes it much more difficult for this class of drugs to discriminate the effects of individual neurotransmitter systems on cytokine production. However, desipramine, a potent NE antidepressant, inhibits TNF-α production and increases the release of IL-10, shifting the balance in favor of Th2 response [366].

Finally, among MonoAmine Oxidase Inhibitors (MAOIs), phenelzine (an irreversible inhibitor of MAO-A and MAO-B) decreases TNF-α production [367], moclobemide (a reversible inhibitor of MAO-A) reduces the production of TNF-α and IL-8 and increases the production of IL-10 [368], and selegiline (a reversible inhibitor of MAO-B) reduces the production of TNF-α and stimulates the biosynthesis of IL-6 and also of IL-1β [369]. MAO-A inhibition is more closely associated with an increase in noradrenergic than in serotonergic system [370], while MAO-B inhibition seems to mainly enhance dopaminergic system [371]; so, in general, MAOIs could shift the cytokine balance towards a Th2 response in relation to the enhancing of catecholamine release.

## ANTIDEPRESSANTS AS IMMUNOMODULATORS

In order to conclude, it can be useful to make a nod to some of the currently available data on literature regarding the experimental use of antidepressants as immuno-modulators or their possible immunomodulatory effects in the course of internistic pathologies.

### Antidepressants use in Inflammatory and Immune-mediated Diseases

Limited data have shown a reduction of Crohn’s disease activity during treatment with bupropion [372, 373], possibly due to TNF decrease [348], and phenelzine [374], possibly related to β adrenoreceptor agonism due to NE increase [348]. Mirtazapine is not recommended, because of the related TNF increase [348], amitriptyline does not appear to be effective [375], whereas data on paroxetine are controversial [376] (one study reported an activation of Crohn’s disease after paroxetine treatment [377]). 

In a study on non-depressed patients with psoriasis, bupropion proved to be effective in reducing skin lesions, as it showed an effectiveness, albeit less incisive, in patients with atopic dermatitis [378]; also MAOIs have shown an efficacy in these conditions [379]. Although SSRIs are generally considered safe in the treatment of psychiatric disorders encountered in dermatological diseases [380], some cases of induction or worsening of psoriasis have been reported during treatment with paroxetine and fluoxetine [381, 382], in which a specific influence of immuno-modulation by 5-HT is assumed [382].

The effectiveness of bupropion and nortriptyline was reported in patients with COPD, a disease associated with cigarette smoking and often depression [383, 384]: nortriptyline treatment was also accompanied by marked improvements in certain respiratory symptoms [384]. Asthma is rather frequently associated with anxiety disorders such as panic disorder [385], for which SSRIs are the treatment of choice [385, 386]; it was noted that some respiratory parameters deteriorated in patients with panic attacks who suspended SSRIs [387], whereas a case of developing of asthma-like reaction during venlafaxine treatment was reported [388]. In this regard, it is noteworthy that glucocorticoid and β2 receptor agonist therapy reduces IL-12 production by antigen presenting cells and greatly suppresses Th2 cytokine synthesis in activated, but not resting T cells, and abolishes eosinophilia; if, however, resting (cytokine-uncommitted) T cells are subsequently activated by antigen presenting cells preexposed to glucocorticoids and β2 receptor agonists, enhanced IL-4 production, but limited IFN-γ synthesis, could be induced, enhancing Th2 response [389]. Thus, while in short term the effects of glucocorticoids and β2 receptors agonists may be beneficial, their long term effects might be to sustain the increased vulnerability of the patient to the allergic condition [1].

In murine T-cell-mediated demyelinating disease model of multiple sclerosis, venlafaxine significantly ameliorated clinical symptoms of the disease in association with a reduction of IL-12, TNF-α and IFN-γ [306]. Treatment with lofepramine (a potent and selective NE uptake blocker [390]) and L-phenylalanine was found to be effective in relieving symptoms of multiple sclerosis, such as pain and fatigue, and in reducing lesion number visible on MRI, hypothetically in relation to noradrenergic activation [391, 392].

MAOIs seem to be effective in rheumatoid arthritis [393, 394], however, SSRIs also significantly inhibited disease progression in an animal model (fluoxetine showing the greatest degree of efficacy at the clinical and histologic levels) [395].

Use of phenelzine and other MAOIs has also been proposed in Behcet’s disease [396] and IgA nephropathy [397].

In animal models of Alzheimer disease, imipramine significantly prevented memory deficit, inhibited the TNF-α increase in frontal cortex and decreased the elevated levels of β-amyloid both in frontal cortex and in hippocampus [398]; prophylactic paroxetine treatment decreased Alzheimer disease like pathology in the animal model [399] and fluoxetine reduced cognitive decline in patients with mild cognitive impairment [400]. Improvement in memory and cognition might be due to hippocampal neurogenesis induced by antidepressants, but the anti-inflammatory properties of antidepressants may be still involved since the inflammation associated with activated microglia has been demonstrated to suppress hippocampal neurogenesis in adult rat [401]. In a mouse model of Parkinson’s disease, paroxetine treatment prevented degeneration of nigrostriatal DA neurons, in association to increased striatal DA levels and reduced expression of IL-1β and TNF-α by activated microglia [402].

### Antidepressants use in Metabolic and Cardiovascular Diseases

Emerged evidences suggest that MAOIs such as selegiline and phenelzine do have some anti-atherogenic effects [403, 404]. Also, reboxetine treatment showed beneficial effects on several metabolic parameters [405]. On the other hand, SSRIs instead seem to induce some clinical and biochemical manifestations of metabolic syndrome, such as insulin resistance [406], hypercholesterolemia and visceral obesity [407]. In this respect it should be noted that the increase in plasma 5-HT related to excessive dietary tryptophan may be atherogenic [408]. However, data on lipid dysregulation in depression indicate that, generically, the treatment of this clinical condition would result in an improved metabolic profile [409]. Furthermore, the use of agomelatine in the metabolic syndrome, by virtue of melatonin properties on preventing a number of sequelae in animal models of metabolic syndrome has been proposed [410], considering that melatonergic agonism seems to reorganize the cortisol secretion [356].

Several data show generic efficacy of antidepressants use, including SSRIs, SNRIs, bupropion, mirtazapine and tricyclics, in patients with coronary artery disease and after myocardial infarction, although certain classes of antidepressants are associated with a higher rate of adverse events [411-413]. SSRIs appear to reduce cardiovascular mortality in acute coronary syndromes, especially when associated with depression [414, 415], whereas tricyclics may be related with cardiovascular negative effects such as arrhythmic activity and increased heart rate [415], and bupropion has been associated with myocardial infarction in isolated cases [416-418]. Beyond these general considerations, it is noteworthy that a recent study on patients with heart failure showed lower levels of TNF-α and CPR in patients treated with tricyclics and SNRIs compared to those treated with SSRIs or those without depression, suggesting that the type of antidepressant used may have a significant effect on the underlying inflammatory process of heart failure [419].

Several antidepressants, particularly those with predominantly NE component such as reboxetine, atomoxetine, nortriptyline, venlafaxine, selegiline, amphetamines, and also some SSRIs, have shown benefits in post-stroke recovery (for example, motor function and depression) probably due to the role of NE in enhancing cortical plasticity [420-427], even if it might be also related to the modulation of the underlying inflammatory process.

### Antidepressants use in Chronic Infections

In HIV infection models, SSRIs significantly decreased HIV viral replication, suggesting an enhancement of natural killer/CD8 HIV suppression [428, 429]; the use of SSRIs as an adjunctive therapy has also been attempted in the treatment of HIV-infected individuals [430]. In addition, mirtazapine has shown in isolated cases some benefit in the treatment or prophylaxis in HIV patients with progressive multifocal leukoencephalopathy [431, 432]. Also hypericin, another main 5-HT enhancing drug [433], possesses high toxicity towards HIV, as well as towards tumors [434]. On the other hand, MAOIs, such as selegiline, and addictive substances, that increase DA availability, seem to enhance viral replication and infection-related neuronal ultrastructural alterations (spongiform polio-encephalopathy) possibly favoring HIV dementia [435, 436]. SSRIs, particularly paroxetine, are recommended in the treatment of depression in course of HCV infection [437, 438]. In active Mycobacterial disease, it has been proposed that mirtazapine might be the first best preference while bupropion should be avoided [348].

### Antidepressants use in Cancer

An association has been found between the use of SSRIs and risk reduction, in some studies it is more evident than in others, of colorectal cancer [439-441], lung cancer [442] and lymphoma/leukemia [443-445]; although the use of SSRIs may stimulate the secretion of prolactin, a potential breast cancer promoter, clinical studies have refuted an increased risk of breast cancer with SSRIs’ use [446-448]. Furthermore, co-administration of ramelteon (MT1 and MT2 agonist) and fluoxetine to increase IL-2 levels in conditions such as metastatic cancer and HIV infection has been proposed [449]. It has also been observed that hypericin seems to inhibit ovarian cancer cells [450]. Mirtazapine, in addition to representing a promising option for the treatment of cancer-related cachexia and anorexia [451, 452], has been proposed as a potential anticancer agent, both as an adjunctive treatment and as a molecule with direct effects itself, in glioblastoma [453], in osteosarcoma [454] and, more generally, during cancer immunotherapy; the drug-induced TNF increase could have a beneficial effect through the enhancement of antigen driven lymphocyte expansion [348]. Also, melatonin and agomelatine have been shown to inhibit the growth of melanoma cells [455]. On the other hand, an association between tricyclics use and increased risk of lung cancer in clinical studies [442], colorectal cancer in preclinical studies (but not in clinical studies) [439, 441] and melanoma has been observed [456]. In a preclinical study on melanoma, pro-metastatic effects and simultaneously inhibitory actions have been demonstrated on the growth of primary tumor (especially in young animals) of desipramine, a potent NE tricyclics, and to a lesser extent also of fluoxetine, which among SSRIs also enhances NE/DA transmission by 5-HT2C blocking [457]. Imipramine instead, a 5-HT tricyclic, appears to inhibit tumor growth in an animal model of stress-related tumoral process [222]. Finally, MAOIs have demonstrated an anticancer effect in hormone-dependent tumors: in animal models selegiline reduced by systemic way the growth of mammary tumors [458, 459], while clorgiline reduced *in vitro* the growth of advanced prostate cancer (it is noteworthy that a high expression of MAO-A has been identified in high-grade primary prostate cancer) [460].

In summary, literature shows some evidence of immunomodulatory effects of antidepressants; however, specific molecules or classes seem to induce positive immunomodulatory effects in some groups of diseases and possibly negative in others. In diseases such as Crohn's, psoriasis, rheumatoid arthritis and atherosclerosis, in which the pathophysiological process appears to shift towards Th1 response, some drugs such as MAOIs, for example, and bupropion in a few cases, have been used with some benefit on immune imbalance, while other drugs such as SSRIs do not seem to be effective. On the other hand, in diseases such as HIV and some cancers, in which the progression of infection or metastatization seems to correlate with a Th2 switch, drugs such as SSRIs and mirtazapine might favorably affect disease control, while the use of other drugs, such as MAOIs in HIV and possibly tricyclics in neoplastic disease, appears to be associated in some cases with disease progression.

## DISCUSSION

Several human chronic diseases seem to be characterized by a stereotyped multiphasic process resulting from complex interactions between predisposing genetic traits, infections, episodic and chronic activation of the neuroendocrine stress system and oscillations in the Th1-Th2 balance system. Changes in cytokine expression often show a mixed pattern. Nevertheless, in the early stages, Th2 activation is often detected in several immune-mediated diseases, correlated with humoral response and allergic reactions or production of autoantibodies, which in some cases appear to be clinically silent, in other determine clinically significant pathophysiological phenomena. In the late stages, the immune response seems to show in several diseases a switch from Th2 to Th1 pattern, correlated with cell-mediated organ inflammation. Also, chronic activation of the Th2-related stress system seems to lead to a Th1 switch with elevation of both Th1 and Th2 inflammatory cytokines that results in chronic systemic inflammation associated with a cluster of metabolic disturbances and cardiovascular diseases. So, episodic activation of the neuroendocrine stress system due to several causes, including stressful events and intercurrent infections together with genetic polymorphisms and epigenetic factors, affects the Th2 response. Conversely, Th2-to-Th1 switch could be related to chronic activation of stress system associated with genetic-epigenetic abnormalities of systemic feedback mechanisms and/or local inflammatory factors, since stress system tolerates phasic rather than chronic activations [46, 461]: a relative hypoactive stress system with defective central and peripheral components (hypothalamic response, adrenal secretion, etc.) may facilitate or sustain the Th1 shift [31, 82-85, 462]. Finally, intercurrent activations of the stress system (due to environmental or internal stressors) on the basis of chronic Th1 hyperactivation could lead to transitory Th1-to-Th2 switches with relative displacements of the immune reserve, allowing a progression of underlying pathologies such as latent viral infections or tumor metastatization. Thus, while acute environmental stress (at large) is associated with Th2 activation, chronic stress, depending on interactions of genetic, epigenetic, infective, tumoral and environmental factors, could lead to a switch towards chronic Th1 activation (at least partially autonomized), possibly in relation to a relative functional impairment of the NEI stress system, as far as superimposed acute stresses can induce new transitory Th2 switches. In addition to systemic oscillations in the Th1-Th2 balance, compensatory systemic adaptations of the cytokine balance related to local inflammatory alterations in organism districts could result in counter-polar alterations of the Th1-Th2 balance in other districts [1]. The neuroendocrine system appears to affect the balance and dynamic switching of cytokine system; however the flow of information appears to be bidirectional, making it a reason of some observations on the relationship between psychopathological alterations and immunological changes. If on one hand bidirectional relationships could be highlighted between oscillations in the Th1-Th2 balance of immune system and oscillations depression-mania in affectivity associated with neurohormonal changes, on the other hand, it is possible that chronic activation of the stress system with relative Th2-to-Th1 switch might associate in the psychopathology with the transition from anxious dimensions to unipolar depressive dimensions. Therefore, the integrated NEI system could correlate, in a bidirectional way, the oscillations in the immune system with affective and behavioral alterations as epiphenomena of systemic pathologies, making reason of eventual integrated activities of drugs such as antidepressants [463].

By correlating the data on the relationship between neurotransmitters and cytokines with those concerning the relationship between antidepressants and neurotransmission and cytokine balance modulation, it is possible to suppose an interaction between the neurotransmitter balance and the Th1/Th2 balance, as basis of the adaptive response of the organism to the environment in human physiology and pathophysiology, whereas the neuroendocrine and immune systems are considered the two major adaptive systems of the body [464]. In the mechanism of action of several antidepressants a 5-HT/NE neuromodulatory balance seems to play an important role, which in turn could influence the Th1/Th2 cytokine balance toward Th1 or Th2 pattern responses. Specifically, it could be hypothesized that as the specific antidepressant increases NE tone, it increases Th2 cytokines production, as it reduces NE tone (or increases 5-HT tone, in turn possibly reducing NE tone), it reduces Th2 cytokines production, shifting the Th1/Th2 balance relatively in favor of Th2 or Th1 response, respectively. Although some data seem at large to correlate neurotransmitter balance with cytokine balance, and in particular the noradrenergic system with the Th2 response [1], it is likely that tentative concept is too simplistic. It is possible that antidepressants do not affect the neurotransmitter and Th1/Th2 balance modulation in an absolute way, but rather in a relative one, ideally compensating a previous imbalance. Antidepressants appear to cause a modulation of neurotransmission and receptor sensitivity with knock-on effect, possibly leading to desensitization of hypersensitive receptors and to hypersensitization of desensitized receptors, thus ideally restoring receptor sensitivity to physiological levels (provided the integrity of neurotransmitter and feedback systems). For example, it has been shown that SSRIs' chronic administration causes a desensitization of 5-HT2A and 5-HT2C receptors, that are found to be hypersensitive in patients with depressive or anxiety disorders [295, 465]. According to this, SSRIs could differently affect cytokine production in different patients who, although classified as depressed by the scales commonly used, could present different symptom clusters related to the alteration of sensitivity of different receptor subtypes. Desensitizing 5-HT1A autoreceptor, SSRIs would mainly increase 5-HT tone and possibly Th1 cytokines; the same SSRIs, however, would also desensitize 5-HT2 receptors, increasing the NE/DA tone and possibly the Th2 cytokines only in those patients presenting with hypersensitivity of these receptors subtypes; thus, different cytokine patterns might depend on neurobiological background of different patients. Likewise, antidepressants would not affect immunomodulation in an absolute way, but would rather compensate in a relative one previous imbalances in Th1/Th2 system. Some studies show that antidepressants can induce differential cytokine changes between responders and nonresponders. In a study on depressed patients, duloxetine increased IL-6 plasma levels only in responders, whereas in nonresponders IL-6 levels remained unchanged; pre-treatment IL-6 levels were lower in those patients who responded to treatment than in those who did not; post-treatment IL-6 levels of responders were normalized by treatment until they reached post-treatment IL-6 levels of nonresponders [318]. Evidences provided by another study gave similar IL-6 results among pre-treatment and post-treatment responders vs. nonresponders, suggesting that IL-6 levels might dichotomize the patients into subsequent responders and nonresponders already at admission [240]. It could be hypothesized that in such cases drugs with high noradrenergic component such as duloxetine lead to clinical response only in depressed patients with a relative hypo-noradrenergic state, as reflected in low basal IL-6 levels (marker of relative deficit of Th2 response); likewise, the same drugs would enhance Th2 response only in those patients with cytokine balance relatively shifted towards Th1 because of Th2 deficit, but not in an absolute way in patients with normal Th2 response or in subjects with compensated Th1/Th2 balance [318]. 

Several factors therefore seem to affect immuno-modulation, including the pharmacologic one. Among these, the timing in the use of immunomodulators, possibly including antidepressants, could play an important role, according to temporal phases and regional distributions of cytokine balance in the Th1-Th2 dynamic switching and its neurohormonal control.

The considerations presented in this work are intended as preliminary, since most of the reported clinical data are based on small samples studies or on individual observations, while for most of the molecules, studies on uniform and large samples and for adequate times are currently the exception. Moreover, in some cases data are consistent, in other cases less concordant; in this regard, it is to be noted the importance of discussion of clinical studies compared to preclinical (e.g. variations in cytokine levels during 52 week study on depressed patients treated with SSRIs [252] compared to those detected in course of some preclinical studies on 5-HT and cytokines interactions [33]). This also happens because *in vitro* effects of molecules on cytokine production can have opposite results depending on the dose and according to the time of action [32]. Considering that antidepressant treatment needs at least 10-14 days for any clinical effectiveness to appear, the treatment *in vitro* cells with antidepressants for 16-24 h appears to reflect only acute effects of the drugs; furthermore, the concentrations used in some *in vitro* studies seem to be rather higher than clinically relevant concentrations [162]. Several other factors could play an important role, such as systemic mechanisms of mutual influence between neurotransmitter and cytokine systems, receptor sensitivity modulation and feedback effects, that may vary in healthy subjects or in patients affected by different pathologies. For example, opposite results on noradrenergic tone in relation to time of action and receptor sensitivity during NRIs administration have been reported [323], as well as opposite results on Th2 cytokines synthesis have been observed in activated and resting T cells exposed to β2 agonists [1]. 

Despite broad limits make it difficult to reach conclusions, overall considerations on neuro-immuno-modulation could represent an additional aid in the study of pathophysiology of psychiatric disorders and in the choice of specific antidepressant (and virtually other drugs) in specific clusters of symptoms especially in comorbidity with several internal pathologies. Finally, antidepressants represent promising immunomodulator agents in psychiatric and non-psychiatric diseases, such as cancer and others. Overall, the reported considerations are tentative and require experimental confirmation or refutation by future studies.

## Figures and Tables

**Table 1.** Neurotransmitters and Cytokines

Neurotransmitters	Receptors	Cytokines	Th1/Th2 Balance Hypothetic Shifts	References
Norepinephrine	β2 adrenoreceptors	↓IL-12, IL-1, IFN-γ, TNF-α ↑IL-10, IL-4, IL6	Th2 shift	Elenkov, 2008 [1]
Serotonin	5-HT1 receptors	↑IL-1β, IFN-γ, TNF-α Conflicting data	Th1 shift	Kubera *et al*., 2005 [32] Conflicting data
Dopamine	D1-like receptors	Th2 differentiation in CD4 naïve Conflicting data	Th2 shift	Nakano *et al*., 2009 [39] Conflicting data
Acetylcholine	N ACh receptors	Upregulation Th1 transcription factors	Th1 shift	Kikuchi * et al*., 2008 [42]
Histamine	H1 and H2 receptors	↓IL-12, TNF-α, IFN-γ ↑IL-10, IL-6	Th2 shift	Elenkov, 2008 [1]
Glucocorticoids	Cytoplasmic/nuclear GR	↓IL-12, IL-1, IFN-γ, TNF-α ↑IL-10, IL-4, IL-13	Th2 shift	Elenkov, 2008 [1]
Melatonin	RZR/ROR MT receptors	↑IL-2, IFN-γ, IL-6 / ↓IL-10	Th1 shift	Kuhlwein and Irwin, 2001 [43] Garcia-Maurino *et al*., 1997 [44]

The table summarizes cytokines modulation, receptors involved and hypothetical influence on Th1/Th2 balance for each neurotransmitter. (IL: Interleukin; IFN: Interferon; TNF: Tumor Necrosis Factor).

**Table 2.** Antidepressants, Neurotransmission and Cytokines

Antidepressants	Pharmacodynamics	Neuromodulation	Cytokines	Th1/Th2 Balance Hypothetic Shifts	References
SSRIs: Paroxetine, Sertraline, Fluoxetine, Escitalopram	SERT inhibition (+ M1 antagonism: paroxetine; 5HT2C antagonism: fluoxetine; DAT inhibition: sertraline)	Desensitization of somatodendritic 5-HT1A autoreceptor (± 5-HT2(A/C) receptors) → 5-HT firing enhanced + NE firing reduced + cortisol decreased	↓IL-4, IL-10, IL-13, IL-6 / ↑IL-1β, IFN-γ, ↑↓IL-2 (Conflicting data)	Th1 shift	Hernandez *et al*., 2008 [252] (others)
SNRIs: Venlafaxine	SERT inhibition ± NET inhibition (only at high dose)	Desensitization of somatodendritic 5-HT1A autoreceptors (± 5-HT2(A/C) receptors) → 5-HT firing enhanced ± desensitization of α2 terminal receptors (± hypersensitization of α1 and desensitization of β1 receptor) → NE firing enhanced (i.e. in PFC and hippocampus) and DA release enhanced in PFC (only at high dose)	↓IL-12, TNF-α, IFN-γ / ↑IL-10, TGF-β1 / ↓IL-6 (low dose) ↑IL-6 (high dose)	Th1 shift (low dose) Th2 shift (high dose)	Kubera *et al*., 2001 [303] (others) Vollmar *et al*., 2008 [305] Kubera *et al*., 2004 [309]
SNRIs: Duloxetine	SERT inhibition + NET inhibition	Desensitization of somatodendritic 5-HT1A autoreceptors (± 5-HT2(A/C) receptors) → 5-HT firing enhanced + desensitization of α2 terminal receptors (± hypersensitization of α1 and desensitization of β1 receptor) → NE firing enhanced (i.e. in PFC and hippocampus) and DA release enhanced in PFC	↑IL-6	Th2 shift	Fornaro *et al*., 2011 [318]
NRIs: Reboxetine	NET inhibition	Desensitization of α2 terminal auto- and heteroreceptor (± hypersensitization of α1 and desensitization of β1 receptor) → NE firing enhanced (i.e. in PFC and hippocampus), DA release enhanced in PFC and 5-HT firing enhanced + cortisol increased	↑IL-10 / ↓IL-1β, TNF-α, IL-6	Th2 shift	McNamee *et al*., 2010 [319] O’Sullivan *et al*., 2009 [322] (others)
NDRIs: Bupropion	NET inhibition + DAT inhibition (+ NACh receptors antagonism)	Desensitization of α2 terminal auto- and heteroreceptor (± hypersensitization of α1 and desensitization of β1 receptor) → NE firing enhanced (i.e. in PFC and hippocampus) and DA release enhanced in PFC and in nucleus accumbens	↑IL-10 / ↓TNF-α, IFN-γ, IL-1β	Th2 shift	Brustolim *et al*., 2006 [336]
Atypical: Mirtazapine	α2, 5HT2, 5HT3, H1 receptors antagonism	Desensitization of α2 terminal heteroreceptor → 5-HT firing enhanced (75%) only at 5-HT1-mediated transmission + NE (30%) and DA release enhanced in PFC + melatonin increased and cortisol decreased	↑TNF-α ↓IL-6 / ↓IFN-γ ↑IL-4	Th1 shift	Kraus *et al*., 2002 [342] Kubera *et al*., 2006 [343]

The table summarizes mechanisms of action, neuromodulatory effects, cytokines modulation and hypothetical influence on Th1/Th2 balance for each class of antidepressants. (SSRIs: Selective Serotonin Reuptake Inhibitors; SNRIs: Serotonin Norepinephrine Reuptake Inhibitors; NRIs: Norepinephrine Reuptake Inhibitors; NDRIs: Norepinephrine Dopamine Reuptake Inhibitors; 5HT: Serotonin; NE: Norepinephrine; DA: Dopamine; H: Histamine; ACh: Acetylcholine; SERT: Serotonin Transporter; DAT: Dopamine Transporter; NET; Norepinephrine Transporter; IL: Interleukin; IFN: Interferon; TNF: Tumor Necrosis Factor; TGF; Tumor Growth Factor; PFC: Prefrontal Cortex)
